# Cytochrome P450 2B Diversity and Dietary Novelty in the Herbivorous, Desert Woodrat (*Neotoma lepida*)

**DOI:** 10.1371/journal.pone.0041510

**Published:** 2012-08-22

**Authors:** Jael R. Malenke, Elodie Magnanou, Kirk Thomas, M. Denise Dearing

**Affiliations:** 1 Department of Biology, University of Utah, Salt Lake City, Utah, United States of America; 2 Laboratoire ARAGO, Université Pierre et Marie Curie-Paris6, Banyuls-sur-Mer, France; 3 Biologie Intégrative des Organismes Marins, Centre National de la Recherche Scientifique, Banyuls-sur-Mer, France; 4 Department of Internal Medicine, University of Utah School of Medicine, Salt Lake City, Utah, United States of America; University of Arkanas, United States of America

## Abstract

Detoxification enzymes play a key role in plant-herbivore interactions, contributing to the on-going evolution of ecosystem functional diversity. Mammalian detoxification systems have been well studied by the medical and pharmacological industries to understand human drug metabolism; however, little is known of the mechanisms employed by wild herbivores to metabolize toxic plant secondary compounds. Using a wild rodent herbivore, the desert woodrat (*Neotoma lepida*), we investigated genomic structural variation, sequence variability, and expression patterns in a multigene subfamily involved in xenobiotic metabolism, cytochrome P450 2B (*CYP2B*). We hypothesized that differences in *CYP2B* expression and sequence diversity could explain differential abilities of woodrat populations to consume native plant toxins. Woodrats from two distinct populations were fed diets supplemented with either juniper (*Juniperus osteosperma*) or creosote bush (*Larrea tridentata*), plants consumed by woodrats in their respective desert habitats. We used Southern blot and quantitative PCR to determine that the genomic copy number of *CYP2B* in both populations was equivalent, and similar in number to known rodent copy number. We compared *CYP2B* expression patterns and sequence diversity using cloned hepatic *CYP2B* cDNA. The resulting sequences were very diverse, and clustered into four major clades by amino acid similarity. Sequences from the experimental treatments were distributed non-randomly across a *CYP2B* tree, indicating unique expression patterns from woodrats on different diets and from different habitats. Furthermore, within each major *CYP2B* clade, sequences shared a unique combination of amino acid residues at 13 sites throughout the protein known to be important for CYP2B enzyme function, implying differences in the function of each major *CYP2B* variant. This work is the most comprehensive investigation of the genetic diversity of a detoxification enzyme subfamily in a wild mammalian herbivore, and contributes an initial genetic framework to our understanding of how a wild herbivore responds to critical changes in its diet.

## Introduction

The interactions between plants and their herbivores represent a large and important fraction of the relationships in any ecosystem. Plants have many weapons in their arsenal of anti-herbivore defenses, including physical, temporal and chemical defenses [1]. To combat the diversity of chemical defenses, vertebrate herbivores rely heavily on enzymatic biotransformation in the liver. A number of enzymes in the liver metabolize plant secondary compounds (PSCs) and other xenobiotics by modifying them to more easily excretable compounds by increasing the polarity through the addition of functional groups or endogenous conjugates. These hepatic biotransformation (“detoxification”) enzymes are promiscuous; although they perform a specific chemical function, they are not specific to a single substrate and react with many dietary toxins [2], [3]. It is likely that the diversity, capacity and functionality of the liver biotransformation system evolved, in part, as adaptive strategy to cope with the large variety of PSCs encountered in nature [4].

Many detoxification enzymes are produced from large families of genes that have arisen from multiple duplication events [5], [6], [7]. These multigene families have the same mechanisms for evolutionary change as single-copy genes, including mutation and regulatory changes. However, duplication events also create genetic redundancy in which nucleotide changes can gradually accumulate or large-scale mutations occur, such as unequal crossing over or gene conversion. Broadly, gene duplication events have four potential functional outcomes: gene conservation, neofunctionalization, subfunctionalization, and pseudogenization [8]. Gene conservation, in which the new copy is identical to the old, may result in protein dosage differences as more product is made. Neofunctionalization is the evolution of new function in the duplicate while subfunctionalization is the division of the ancestral gene's functions between the two daughter copies. Pseudogenization via loss-of-function mutations will not affect the function of the original gene copy, which theoretically persists elsewhere in the genome in its original state [9]. As a result, the process of duplication followed by genetic diversification may be an especially effective mechanism for adaptive evolution. Indeed, there is evidence that recently duplicated genes are more likely to experience positive selection than equivalent single copy genes [10], emphasizing the power of gene duplication for the promotion of adaptive evolution.

The process of duplication followed by genetic diversification may be an especially effective mechanism for groups such as detoxification genes that face a wide variety of substrates like PSCs. The cytochrome P450 superfamily (P450) is a large, multigene family with at least 20 genes in mycobacteria and, on average, 50–100 genes in vertebrate species, e.g., there are 57 P450 genes in humans [11], [12], [13]. Many of the enzymes produced by P450 genes are associated with biotransformation (Phase 1) of xenobiotics in the liver [14], [15]. This diversity is due, in part, to a history of repeated duplication events as well as gene conversions and lateral transfers [12], [16], as might be predicted of genes selected for dealing with diverse plant toxins. In an analysis of 10 vertebrate genomes, Thomas [17] found that cytochrome P450 genes involved with xenobiotic metabolism were more likely to undergo duplication events than P450 genes involved with endogenous compound metabolism. In insect herbivores, genetic divergence in P450 genes has been correlated with the ingestion of novel dietary toxins [18], even to the level of single amino acid changes affecting substrate-processing rates [19].

In mammals, proteins of the P450 gene families, “CYP” 1, 2, and 3 metabolize drugs and other xenobiotic compounds such as environmental contaminants [11]. As a result, in the field of pharmacology, the diversity and function of the proteins made from these gene families have been studied extensively using model mammal species (rat, mouse, dog, rabbit). It is likely that proteins from these P450 families also play a critical role in the metabolism of PSCs by wild mammalian herbivores. Basic genetic information for these genes would be valuable for understanding the detoxification process in wild herbivores yet P450 sequence data from wild herbivores are restricted to a handful of species, many of which are marsupials [20]. Marsupials are exemplary for the study of PSC metabolism because many species consume *Eucalyptus*, whose leaves are heavily defended by phenolics and terpenes. For example, koalas (*Phascolarctos cinereus*) can survive on a diet composed exclusively of *Eucalyptus* and, compared to non-*Eucalyptus* feeding marsupials, have greater expression of *CYP4A15* [20], [21], [22]. Despite this association, the molecular characterization of P450 enzymes in marsupials and eutherian herbivores is relatively unknown. The role of P450s in metabolizing xenobiotic compounds suggests that the neofunctionalization and subfunctionalization (as mechanisms for specialization [8]) of duplicate genes might be positively selected for in herbivores dealing with a broad and toxic diet. However, little has been documented of allelic diversity of P450 genes within wild species, and even the number of loci in important detoxification subfamilies remains unknown.

To advance our understanding of the detoxification system of wild herbivores, we conducted a genetic study of the *CYP2B* subfamily in two populations of a wild herbivore, *Neotoma lepida* Thomas (the desert woodrat). In vertebrates, the CYP2 family is the largest P450 family, having undergone multiple duplication events in different lineages [13], [23]. The CYP2B subfamily in this group is associated with xenobiotic detoxification; in humans, the CYP2B subfamily is responsible for the metabolism of more than 25% of commercial drugs [24]. In addition, in rodents and marsupial lineages, *CYP2B* has duplicated more frequently than other subfamilies in other vertebrates [13], [25]. Even closely related species, such as Norway rat, *Rattus norvegicus*, and house mouse, *Mus musculus*, have unique *CYP2B* duplication histories. Within a species, isoforms (transcripts of paralogous genes) of *CYP2B* may have up to 97% nucleotide similarity in coding regions but may have different substrate affinities [6]. Despite the associations between CYP2B function and diversity in pharmaceutical or medical studies, little is actually known about the genetic mechanisms of dietary adaptation in wild herbivores.

Desert woodrats (*Neotoma lepida*) present a natural experiment in which to investigate the detoxification strategies used by wild mammalian herbivores to deal with the toxic challenges. At the end of the Pleistocene (18,700–10,000 ya), creosote bush (*Larrea tridentata*) invaded the North American southwest from South America [26]. The range of creosote bush expanded as temperatures warmed, and juniper (*Juniperus spp*.), which had been widespread, was pushed northward and to the higher elevations of the Great Basin Desert [26]. This change in habitat resulted in a change in available forage for local herbivores. Evidence from fossilized desert woodrat middens in the Mojave Desert suggests that woodrats stopped collecting juniper and started collecting creosote bush at this time [27]. Creosote bush and juniper have very different suites of PSCs [28], [29], and this diet switch resulted in new biotransformation challenges for *N. lepida*. Currently, woodrats from the Mojave Desert are able to consume more creosote resin with fewer harmful effects than Great Basin woodrats [30], [31]. Previous work has linked the differential ability of Mojave and Great Basin Woodrats to metabolize creosote bush PSCs to CYP2B [32], [33], [34]. Desert woodrats from the Mojave Desert have constitutively higher CYP2B activity but not more protein expressed, compared to animals from the Great Basin Desert [32]. The difference in CYP2B activity but not protein content of the Mojave woodrats indicates there may be a genetic change in the CYP2B enzyme of Mojave animals, perhaps in response to their evolutionary history with creosote bush.

The overall goal of this work was to investigate the evolutionary mechanisms used by a wild herbivore to process the arsenal of plant chemical defenses in natural diets. We explored the potential for genetic diversity in the *CYP2B* subfamily to explain how Mojave Desert woodrats are able to ingest greater quantities of creosote resin than Great Basin woodrats. We hypothesized that Mojave Desert woodrats had unique CYP2B enzymes that contribute to the metabolism of creosote. These unique enzymes could result from allelic diversity or from differences in the number of *CYP2B* copies (isoforms) in Mojave Desert versus Great Basin animals. The alternative hypothesis is that, rather than rely on unique, substrate-specific enzymes, Mojave Desert woodrats have the same suite of *CYP2B* variants as Great Basin animals, but rely on distinctive expression patterns to optimally metabolize creosote bush PSCs. To address these hypotheses, we evaluated *CYP2B* gene copy number in Mojave and Great Basin woodrats, comparing them to known *CYP2B* loci numbers in other rodents. We also generated the first full-length cDNA sequences of woodrat *CYP2B* and compared expression and amino acid make-up of *CYP2B* variants across treatment groups. We discovered potentially significant amino acid changes at important substrate recognition sites known to result in functional differences in pharmacological studies of CYP2B enzymes.

## Methods

### Ethics statement

All procedures in this experiment were approved the University of Utah Institutional Animal Care and Use Committee (IACUC #07-02015).

### Woodrat samples

Archived *N. lepida* livers and spleens were used to compare woodrat detoxification strategies. The samples were from an experiment comparing gene expression profiles between two populations of *N. lepida* on diets supplemented with two different natural suites of PSCs [34]. The woodrat individuals were trapped from two distinct population of *N. lepida* in the Great Basin Desert (Tooele Co., UT) and the Mojave Desert (Washington Co., UT). In the lab, five individuals from each population were fed a rabbit chow based diet (Harlan Teklad formula 2031) supplemented with either juniper foliage or a resin extracted from creosote bush foliage. Juniper is presumed to be an important component of the ancestral diet of the Mojave woodrats [27], [34] and is currently included in the diets of Great Basin woodrats [30]. In contrast, creosote bush is an important component in the current diet of Mojave woodrats and is novel to Great Basin woodrats [35]. Both populations responded similarly to the juniper diet, consuming similar amounts of juniper and ending the trial in equivalent negative mass balance. However, on the creosote bush diet, Mojave Desert woodrats consumed more creosote resin and maintained positive mass balance, whereas Great Basin Desert woodrats consumed less resin and ended in negative mass balance. [For details on experimental design, diet preparation or feeding trial results, see Magnanou et al. 2009.]

After the feeding trial, animals were dispatched using CO_2_ asphyxiation; liver tissue and spleens were frozen with dry ice or preserved in RNAlater (Sigma) and archived at −80°C. For Southern Blotting and quantitative PCR, DNA was extracted from woodrat spleens and treated with RNase-A. For *CYP2B* cDNA cloning and sequencing, RNA was extracted from the livers, treated with DNase and converted to cDNA (Applied Biosystems).

### Woodrat *CYP2B* characterization

Genbank was searched for *CYP2B* cDNA sequences for a variety of isoforms from Norway rat, *Rattus norvegicus*, and mouse, *Mus musculus* (Table 1). The last common ancestor of these species and the genus *Neotoma* existed 22–25 mya [36]. Primers were designed using this alignment and subsequent *Neotoma* sequences. The full open reading frame was captured using rapid amplification of cDNA ends (First Choice RLM-RACE kit, Ambion; Table 2 for primer sequences). The resulting sequence was BLASTed against Genbank to compare it to existing sequences.

**Table 1:** Rat (*Rattus norvegicus*) and mouse (*Mus musculus*) *CYP2B* isoforms.

Species	*CYP2B* isoform	Genbank #
*M. musculus*	2b9	NM_010000.2
*M. musculus*	2b10	AK028103.1
*M. musculus*	2b13	NM_007813.1
*M. musculus*	2b19	NM_007814.1
*M. musculus*	2b23	NM_001081148.1
*R. norvegicus*	2b1	XM_001070869.1
*R. norvegicus*	2b3	NM_173294.1
*R. norvegicus*	2b12	NM_017156.1
*R. norvegicus*	2b21	NM_198733.1
*R. norvegicus*	2b31	XM_577774.1

**Table 2:** Woodrat primer names, sequences and uses.

Primer Name	Sequence (5′to3′)	Use
5′RACE Outer (forward)	GCTGATGGCGATGAATGAACACTG	Initial sequencing: 5′ end amplification
Rodent Outer (reverse)	A(G/A)GAACTGG(T/C)GGTCTTTGT	
5′RACE Inner (forward)	CGCGGATCCGAACACTGCGTTTGCTGGCTTTGATG	
Rodent Inner (reverse)	GATGATGTTGGCTGTGATGC	
NL_CYP2B_L3 (forward)	GCATCACAGCCAACATCATC	Amplification of 3′ end
3′RACE_outer (reverse)	GCGAGCACAGAATTAATACGACT	
NL_CYP2B_L4 (forward)	CCCCATGTTGCAGAGAAAGT	
NL_CYP2B_L4 (forward)	CGCGGATCCGAATTAATACGACTCACTATAGG	
NL_CYP2B_L6 (forward)	GYCASACCAGGACCAYGGRG	Full-length CYP2B ORF in *N. lepida*
NL_CYP2B_H7 (reverse)	GACACCTGGCCACCTCAG	
NL_CYP2B_L3 (forward)	GCATCACAGCCAACATCATC	To sequence internal read from amplified ORF
NL_CYP2B_L8(forward)	GAAAGCGCATTTGTCTTGG	Amplification of probe for Southern Blot
NL_CYP2B_H9 (reverse)	GTATAGGCAAGTCCATAGACA	
NL_CYP2B_L10 (forward)	CTCTCCTCGTGGRCTTTGTA	qPCR primers unique to *N. lepida* sequences
NL_CYP2B_H10 (reverse)	AGGAARCCTCCTCTGTCCAT	
NL_CYP2B_L11 (forward)	CCCAYCCTGAGTTCAGCTCT	qPCR primers conserved across *N. lepida*, rat, mouse.
NL_CYP2B_H11 (reverse)	TTTCTTCAGTGCCCCATTG	
NL_SOD (forward)	GAGACCTGGGCAATGTGACT	qPCR primers for single copy control
NL_SOD (reverse)	CAATGATGCAATGCTCTCCT	

### Gene copy number

We used Southern blotting to estimate *CYP2B* gene copy number in Great Basin and Mojave woodrats. To create a large enough probe (∼550 bp) of continuous sequence, we used the final exon (182 bp) plus part of the 3′UTR (367 bp) of the *CYP2B* message. We amplified this region from the genomic DNA of one Great Basin woodrat and cloned it into a pJET1.2/blunt vector (Fermentas). Successful clones were sequenced to verify capture of the insert. To ensure a pure product, the fragment was PCRed a second time using a single cloned product. The purified PCR product was labeled with [32P]dCTC and used as the probe. DNA from two experimental animals from each population (Great Basin and Mojave) was fragmented with three restriction enzymes: AFLII, BSTEII, and HINDIII. Blots were exposed twice, with additional washing between exposures to remove less tightly bound probe.

We used quantitative PCR on genomic DNA to estimate *CYP2B* copy number [37]. Using woodrat, rat and mouse *CYP2B* sequences, we created two sets of primers to target *CYP2B* isoforms. The first set of primers was designed from a region of *CYP2B* that was conserved within *N. lepida* but divergent compared to rat and mouse sequences. The second set of primers was designed from a region of *CYP2B* that was relatively conserved across woodrat, rat and mouse isoforms (Table 2). We predicted this second primer set would reveal gene copies not captured by the first, woodrat-specific primer pairs or Southern blot. Primers were also designed for superoxide dismutase I (SOD-I), which served as a single-copy gene target to normalize the ratios recovered for *CYP2B* (Table 2).

Quantitative PCR was performed using a Roche Lightcycler 2.1. Each 10 µL qPCR reaction included: 1 µL of mixed forward and reverse DNA oligo primers (5 µM each), 2 µL 5× Master Mix (LightCycler® FastStart DNA MasterPLUS SYBR Green I, Roche), 4.5 µL PCR-grade water and 2.5 µL of DNA (50–100 ng/µL). All reactions were performed in triplicate with samples from both populations. The formation of a single PCR product was confirmed with melting curves and visualization of a single product on 1% agarose gel. Relative copy number ratios were calculated by comparing target *CYP2B* amplification to *SOD-I* amplification.

### Evaluation of CYP2B sequence variation

To estimate the diversity of *CYP2B* messages expressed by desert woodrats in the feeding trial, we cloned and sequenced the ORF from two or three animals in each of four treatment groups from Magnanou et al. (2009). To amplify potentially variable sequences, four ambiguous bases were included in the forward primer that would allow it to bind to 16 combinations of nucleotides (Table 2). Successful PCR products were cloned (TopoTA, Invitrogen) and then sequenced at the University of Utah Core Sequencing Facility and the University of Washington High Throughput Sequencing Facility (UWHTSF) in Seattle, Washington. Because the average read size was smaller at UWHTSF compared to the University of Utah Core Sequencing Facility, an additional forward internal read was added, starting at bp 517. Complementary chromatograms were evaluated in Sequencher 4.5, and amino acid sequences were digitally translated from the cDNA reads [MEGA5 [38]]. Contigs that did not result in a complete, uninterrupted ORF were excluded. The resulting sequences were submitted to Genbank (accession numbers JN105874–JN105960).

Cloning followed by Sanger sequencing was chosen in this situation to recover as many unique *CYP2B* sequences as possible. Different *CYP2B* loci in rat and mouse have been shown to be up to 98% similar, and alleles can vary at a single nucleotide [6]. Because of the high degree of similarity, we could not design quantitative PCR primers that could distinguish between *CYP2B* messages or variants reliably. We also employed next generation sequencing techniques on woodrat liver RNA but 1.5 plates of 454 sequencing did not replicate the diversity of full-length ORF messages we recovered by cloning (Dearing, unpublished data). By some estimates, more than 20 plates of 454 runs are required to reliably cover 75% of the transcripts in the transcriptome of a non-model species for which no genomic framework exists, such as *Neotoma lepida* [39]. The resources were not available to pursue such an extensive next generation sequencing project.

#### Dereplication of sequences

Using the dereplication program FastGroupII [40], we cataloged the number of unique amino acid sequence variants recovered by cloning. FastGroupII is generally used with bacterial 16S environmental datasets to categorize sequences into operational taxonomic units using percent sequence similarity. The advantage of analyses like dereplication is a reduction of the influence of random errors, such as those introduced during reverse transcription and PCR amplification. We used amino acid instead of nucleotide sequences to reveal divisions that might be informative for the function of the protein. We performed dereplication on all 87 of clones, using the “percent identity with gaps” algorithm to group sequences with >95% amino acid similarity.

#### Sequence analysis

To visualize variation and relationships across *CYP2B* sequences, maximum likelihood trees were reconstructed from the nucleotide and putative amino acid sequences. In addition to our woodrat sequences, muroid *CYP2B* sequences were added to the dataset: *Mus musculus CYP2B9*; *M. musculus CYP2B10*; *M. musculus CYP2B13*; *Rattus rattus CYP2B1* and *R. rattus CYP2B3* (Table 1). The entire set of sequences was rooted with *Homo sapiens CYP2B6* (AAF13602.1).

For the maximum likelihood (ML) nucleotide tree, the model of substitution was selected in Modeltest v 3.8 [41] using the Akaike information criterion for each position of the codon. The more complex model was found at the second position (GTR+G), and was applied to all three positions in the ML reconstructions. A ML heuristic search, using a parsimony starting tree, was then conducted using PAUP 4.0b10 package [42] and displayed in Geneious Pro 5.4.6 [43]. 100 bootstrap replicates were performed to evaluate clade robustness.

To reconstruct the amino acid maximum likelihood tree, the substitution model was selected using ProtTest [44]. The best model based on the Akaike information criterion was JTT. It was implemented in PhyML displayed in Geneious. 1000 bootstrap replicates were performed to evaluate the robustness of clades. We also used the amino acid sequences to create a strict consensus tree using parsimony. We conducted searches with 10 random addition replicates with TBR branch swapping, and ran 1000 bootstrap replicates to evaluate the robustness of clades.

To compare the expression of CYP2B variants across experimental treatments, the distribution of the sequences across the maximum likelihood amino acid tree was analyzed using Fast UniFrac [45], a program designed to compare microbial communities from different environments taking into account phylogenetic relatedness of sequences. The distribution of the experimental treatments was considered overall on the tree, with 1000 bootstrapped permutations. Posthoc pairwise comparisons were also performed, correcting (Bonferroni) for the number of pairs compared (n = 6).

Several different approaches were used to explore the roles of positive and negative selection in generating the diversity of *CYP2B* messages sequenced. First, the rates of nonsynonymous and synonymous changes were evaluated across the entire ORF and were tested for significance using a codon-by-codon Maximum Likelihood z-test of neutrality (1000 bootstrap replicates to determine variance; MEGA5). In addition, we searched for signatures of positive and negative selection at each individual codon. Using the HyPhy package [46], [47] to evaluate the same alignment as was used for the phylogenetic reconstruction, putative sites of recombination were assessed with the GARD algorithm, and the two resulting nonrecombinant fragments were analyzed separately with the SLAC codon-based maximum likelihood method. The best time-reversible model was detected and substitution rate parameters were estimated. These values along with branch length (NJ tree) allowed us to obtain a global non synonymous/synonymous substitutions (dN/dS) ratio. Ancestral sequences were then inferred site by site by ML providing the level of significance of positive or negative selection at each codon position of the *CYP2B* ORF. Positive selection was evidenced when dN/dS was significantly greater than 1 (p<0.1) while purifying selection corresponded to dN/dS<1. Otherwise a site was under neutral selection (dN/dS = 1).

We also took a P450 molecule-specific approach by evaluating substitutions at amino acids in conserved substrate recognition sites associated with function [48], [49]. Using the primary pharmacological literature on CYP2B, we identified 13 important amino acid residues within substrate recognition sites in the woodrat sequences (see Table 3 for citations). We mapped the amino acid identity at these important sites across the topology of the CYP2B phylogeny. To evaluate the relative strength of selection for changes in the 13 important amino acid residues, we compared the number of synonymous and non-synonymous substitutions in these 13 codons to the number of synonymous and nonsynonymous changes across the remaining 479 codons of the message.

**Table 3:** Unique combinations of amino acid residues at 13 important substrate recognition sites.

	Important residues from substrate recognition sites:												
	114	206	209	290	294	297	298	302	363	367	477	478	480
**Clade A**	**I**	F	**M**	I	S	**F**	A	T	**A**	**A**	**I**	G	**V**
**A1**	**I**	F	**M**	I	S	**L**	A	T	**A**	**A**	**I**	G	**V**
**B**	**I**	F	**M**	I	S	**F**	A	T	**V**	**V**	**F**	G	**V**
**C**	**I**	F	**M**	I	S	**F**	A	T	**I**	**V**	**F**	G	**V**
**D**	**I**	F	**M**	I	S	**F**	A	T	**A**	**A**	**F**	G	**V**
**E**	**I**	F	**M**	I	S	**F**	A	T	**A**	**A**	**V**	G	**V**
**Clade F**	**I**	F	**I**	I	S	**F**	A	T	**I**	**V**	**F**	G	**L**
**Clade G**	**I**	F	**I**	I	S	**F**	A	T	**V**	**V**	**F**	G	**V**
**Clade H**	**V**	F	**I**	I	S	**F**	A	T	**I**	**V**	**F**	G	**V**
**I**	**I**	F	**I**	I	S	**F**	A	T	**A**	**A**	**I**	G	**V**
**J**	**V**	F	**M**	I	S	**F**	A	T	**I**	**V**	**F**	G	**L**
**K**	**V**	F	**I**	I	S	**F**	A	T	**V**	**V**	**F**	G	**V**
**L**	**V**	F	**I**	I	S	**F**	A	T	**I**	**V**	**F**	G	**L**
**Human 2B6**	**I**	F	**I**	L	S	**F**	A	T	**L**	**V**	**V**	G	**I**
**Rabbit 2B4**	**I**	F	**I**	L	S	**F**	A	T	**I**	**V**	**V**	G	**V**

From the woodrat CYP2B dataset, amino acid residues at 13 sites shown to be important for CYP2B function in model systems [57], [60], [61], [62], [63], [64], [65], [66], [67], [68], [69], [70]. Letters refer to unique combinations of these 13 important residues as they are represented across branches and individual sequences on the CYP2B phylogeny (Figure 3). Columns with bolded amino acid abbreviations indicate residues that vary across the dataset. Corresponding human and rat CYP2B isoform residues are included for comparison.

## Results

### Woodrat *CYP2B* characterization

The cDNA resulting from the *CYP2B* sequencing effort was 1476 bp from start to stop codons (Figure S1). When blasted against the nucleotide database at NCBI, the top hit was to *M. musculus CYP2B10* cDNA with 84% coverage and an e-value of 0.0. When translated, the sequence was 491 amino acid residues long, and also blasted to *M. musculus CYP2B10*.

### Estimating gene copy number

The genomic DNA fragmented by the restriction enzymes was distributed evenly across the length of the gel, and, within a single restriction enzyme treatment, the labeled band patterns were similar for all individuals. There were no consistent differences in the number of bands from Great Basin vs. Mojave animals. The number of gene copies varied with wash stringency. The most conservative estimation of *CYP2B* gene copy was 2; the least conservative estimate for both populations was 7 or 8. We did not compare gene copy number by diet treatment since the genomic background of animals is unaffected by experimental diet.

**Figure 1:**
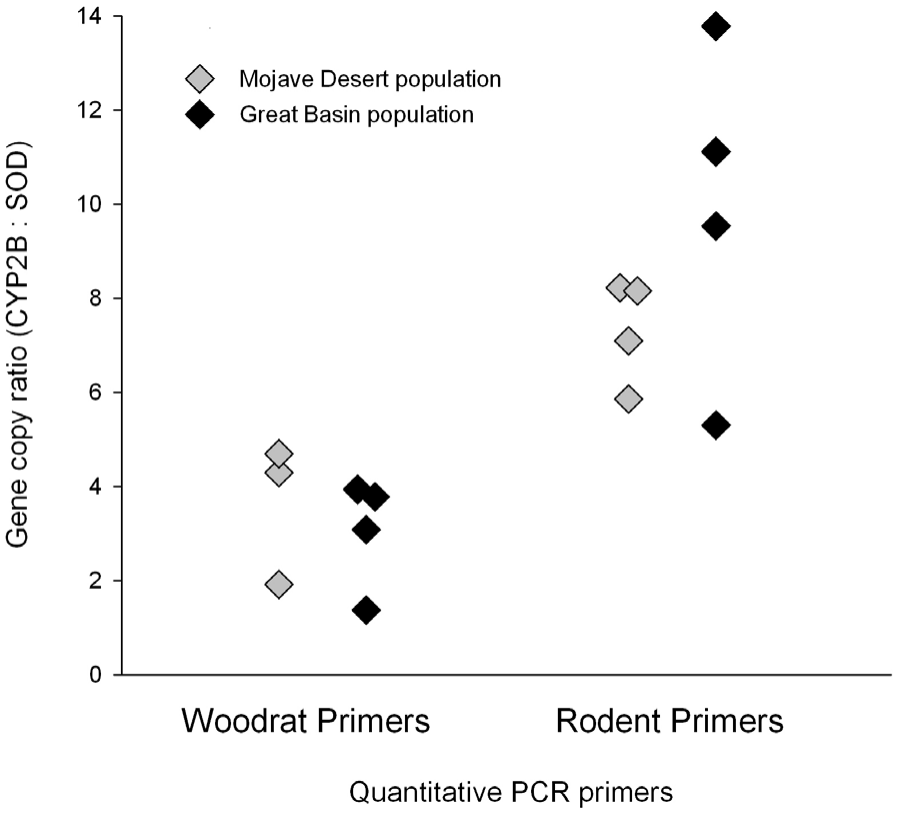
Number of woodrat *CYP2B* gene copies from individuals of both populations using qPCR. Copy number was determined by comparing *CYP2B* amplification to the amplification of the single copy gene, *SOD*-I.

In the qPCR analysis, the primers from the region of *CYP2B* conserved across rat, mouse and woodrat recovered a significantly higher copy number ratio (mean = 8.6±2.8s.d.) compared to the amplified region specific to woodrats (mean = 3.3±1.2s.d.; student's t-test: t = 4.87; df = 9.9; p<.001; Figure 1). However, there was no difference in the copy number recovered by either primer pair for Great Basin and Mojave woodrats (student's t-test; rodent-specific primers: t = −1.397; df = 3.6; p = 0.243; woodrat-specific primers: t = 0.566; df = 3.7; p = 0.604).

### Evaluation of *CYP2B* sequence variation

We sequenced 87 *CYP2B* cDNA clones. The sequences were variable; the average p-distance (the proportion of sites at which any two sequences being compared are different) across the whole dataset was 0.031. This dataset was parsed into 62 unique nucleotide sequences, and 53 unique putative amino acid sequences. The sequencing effort was distributed relatively evenly across the treatments: 27 sequences from Great Basin animals (n = 3) fed juniper, 17 from Great Basin animals (n = 2) fed creosote, 22 from Mojave animals (n = 3) fed juniper and 21 from Mojave animals (n = 3) fed creosote. On average, 7.9 (range: 1, 16) clones were sequenced from each animal: 8.8 (range: 2, 16) for Great Basin animals and 7.2 (range: 1, 14.) for Mojave Desert. The average number of unique amino acid sequences recovered for each animal was 5.3 (range: 1, 12). Great Basin animals averaged 5.8 (range: 2, 12) unique amino acid sequences per individual, and Mojave animals averaged 5.3 (range: 1, 9).

#### Dereplication of sequences

The dereplication of the entire amino acid sequence dataset indicated 3 to 4 different sequence variants at 95% sequence similarity.

#### Sequence analysis

**Figure 2:**
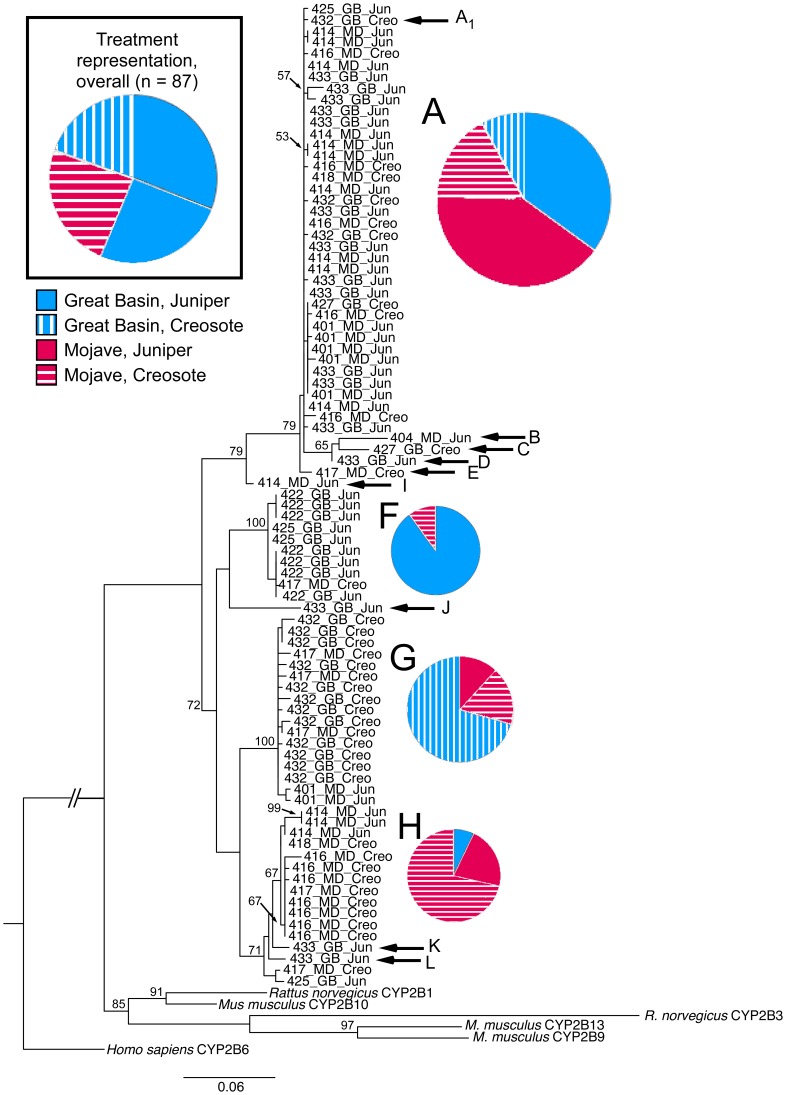
Maximum likelihood tree of woodrat CYP2B amino acid sequences. Branch length is scaled to the amount of differentiation (see scale bar). Bootstrap support of >50% is indicated on the nodes. Branch labels consist of an individual animal identification number followed by population and diet treatment from the feeding trial. The tree is rooted with human CYP2B6 (#AAF13602.1). Letters (A to L) designate individual sequences or clades that share the same combination of amino acids at the 13 sites known to be important for CYP2B function. Letters correspond to the amino acid characterization in Table 1. For the four major clades, tree topology and the unique combination of substrate recognition amino acids are correlated. The pie charts specify the distribution of experimental treatments comprising each unique combination of substrate recognition residues in the major clades (A, F, G, and H). Size is a relative indicator of the number of sequences represented in that pie chart. For comparison, the pie chart in the upper left corner shows the relative representation of experimental treatments in the whole cloned dataset.

The topology of the nucleotide and amino acid maximum likelihood trees were very similar (Figure S2, Figure. 2). Both trees (n = 87 woodrat CYP2B sequences) had four major clades with the same component sequences, and both trees grouped all the woodrat sequences together with the muroid sequences as single sister group, rather than interspersed across the tree. Three of the four major clades have sequences from both the Great Basin and Mojave populations as well as from both dietary treatments, but not necessarily from all four treatment combinations. The amino acid tree reconstructed using parsimony was equally similar in topology (data not shown). Because of these similarities, future analyses and comments will refer specifically only to the amino acid maximum likelihood tree (Figure 2).

Analysis of the distribution of the four experimental treatments revealed that, overall, the treatments were distributed significantly differently across the tree (Unifrac significance test, p = 0.015). In the post-hoc pairwise comparisons, the distribution of the sequences derived from Great Basin animals fed juniper was significantly different from distribution of sequences from Great Basin animals fed creosote resin (p<0.001) and from Mojave Desert animals fed juniper (p<0.05, Unifrac significance tests with Bonferroni corrections).

The global dN/dS ratio for the woodrat CYP2B sequences was 0.54, (with muroid sequences included dN/dS = 0.34) indicating negative or stabilizing selection (dN/dS<1). The codon-by-codon test of neutrality also supported evidence for negative selection (p<0.001, Z = −4.24). Across the woodrat sequences, 302 of the 491 CYP2B codons (including 5 of the 13 important amino acids in known CYP2B substrate recognition sites) displayed no variation across all of the sequences. At 64 of the remaining 189 variable sites, there was evidence for stabilizing selection (dN/dS<1, p<0.1). No codons were significantly under positive selection (dN-dS>1). The results were similar even when the muroid sequences (rat and mouse) were added to the dataset. 85 codons were identical across muroid and *Neotoma* sequences (including 2 of the 13 substrate recognition codons). 64 sites experienced stabilizing selection (p<0.1), and no codons were undergoing significantly positive selection. None of the 13 codons associated with substrate recognition were under negative or positive selection in either analysis.

However, examination of the 13 important amino acids in known CYP2B substrate recognition sites revealed multiple amino acid combinations in the dataset (Table 3). The distribution of these unique combinations corresponded to larger patterns of variation in the phylogeny (Figure 2). In general, clades of sequences, grouped by overall amino acid similarity, also shared the same residues at each of the 13 important sites. In addition, there was a significant difference in the proportion of nonsynonymous (n = 21) to synonymous (n = 6) substitutions in the codons (78% nonsynonymous) composing the 13 residues of interest compared to the nonsynonymous (n = 181) and synonymous (n = 144) substitutions (56% nonsynonymous) of the 479 remaining codons in the cDNA (Fisher's exact; p<0.05).

## Discussion

We used a comparative approach to evaluate the diversity of the *CYP2B* subfamily in creosote bush- and juniper-feeding populations of desert woodrats. In both populations, we found evidence of multiple *CYP2B* gene copies within individuals, and the concomitant expression of more than one gene “variant,” regardless of diet. We did not find evidence of an independent *CYP2B* duplication event in the Mojave woodrats that might explain their ability to better metabolize creosote bush, but we did find that animals in different treatment groups (population×diet) have different patterns of *CYP2B* variant expression with evidence of functional differences. This difference in relative expression may be key to the consumption of creosote bush toxins and may explain why the overall CYP2B enzyme activity of Mojave animals is constitutively higher than Great Basin animals [32].

### *CYP2B* copy number

A synthesis of the three approaches used herein to estimate gene copy number suggests that woodrats have a minimum of three *CYP2B* isoforms and a maximum of eight. Data from the genomic assays indicate woodrats possess anywhere from two to eight *CYP2B* isoforms. The dereplication of the cDNA dataset revealed three to four distinct variants of CYP2B differing from each other at ≥5% amino acid content. This is roughly mirrored in the four major clades of the CYP2B amino acid tree. This degree of differentiation is within the range of CYP2B isoforms in other rodents. For example, the *CYP2B* isoforms 15 and 31 in Norway rat share 97% nucleotide identity over their coding regions, and are 94% identical to *CYP2B12*. These genes represent some of the more recent duplication events in the rat *CYP2B* subfamily [6], so it may be reasonable to assume the woodrat *CYP2B* variants in our sequence dataset are also relatively recently diverged.

The difference between the number of sequence variants in the cDNA dataset (n = 3–4) and the upper range of *CYP2B* loci estimated by qPCR and Southern blot (n = 8) may be indicative of *CYP2B* isoforms that are too divergent to be amplified by the sequencing primers. An upper limit of eight functional isoforms fits within the estimates of *CYP2B* loci in other rodents. There are six to seven functional *CYP2B* genes and four pseudogenes in rat, and five functional *CYP2B* genes and five pseudogenes in mouse [6]. Pseudogenes are unlikely to be present in this sequence dataset since all the sequences are full-length ORFs without evidence of frameshift, but neither qPCR nor Southern blotting require full-length, transcribed cDNA to detect genes. The presence of pseudogenes could be very interesting because it represents the on-going evolution of the *CYP2B* gene family in woodrats.

It is likely that some of the *CYP2B* variants we have detected are not direct homologues of the functional *CYP2B* genes in either rat or mouse but are unique to the *Neotoma* lineage. The common ancestor of primates and rodents (70 mya) had one *CYP2B* gene, while the shared ancestor of family Muridae (encompassing rat and mouse) had 4 *CYP2B* loci (8–10 mya) [6]. Woodrats are in family Cricetidae, which split from the muroid lineage 22–25mya [36]. Thus, the last common ancestor of *Neotoma* and the Murids existed 22–25 mya and, parsimoniously, might have had anywhere from 1–4 copies of *CYP2B*. Conservatively, these historical copy numbers suggest *N. lepida* and the Muridae common ancestor share the same four homologous *CYP2B* loci. However, the topology of the amino acid tree indicates a different hypothesis. All the woodrat sequences form an ingroup, with the muroid and human CYP2B as an external sister group, rather than scattered throughout the woodrat sequences. According to this pattern, it seems likely that the woodrat sequences are more recently and independently evolved than the muroid sequences and not strict homologs. A multi-loci genomic sequencing effort was out of the scope of this project, but would provide the means of testing this hypothesis.

### CYP2B Hypervariation

The sequencing effort revealed a diversity of *CYP2B* messages. We found 62 unique nucleotide sequences that translated into 53 unique amino acid sequences. This variability likely represents a combination of allelic variation at individual loci as well as the sampling of multiple *CYP2B* isoforms. There is significant evidence from human populations that genetic polymorphisms are common in P450 genes [50], [51] including polymorphism at individual loci within the *CYP2* family [52], [53]. For example, there are 19 alleles described for the *CYP2D6* gene [54] and 29 known alleles of the *CYP2B6* gene in humans [55]. These examples document individual SNPs in single *CYP2B* loci using the many genomic tools available for a well-studied organism like humans. These tools are not available for woodrats, and we are almost certainly sequencing more than one *CYP2B* gene, thereby increasing the scope for diversity. At the extreme end, 14 unique *CYP2B* sequences were sampled from a single individual woodrat, which conservatively would indicate seven distinct loci, if the individual was heterozygous at every locus. Such heterozygosity would be rare, and, the variation across some of the gene copies would be so low as to be indistinguishable from allelic differences. In fact, within the 14 unique sequences, 11 are ≥99% similar to each other at the nucleotide level, a value that suggests a combination of allelic variation and processing error. To avoid over-interpreting the single nucleotide or amino acid level variation, we have chosen to focus on the patterns discernable across the more divergent *CYP2B* variants, i.e., the four major clades.

The diversity of CYP2B amino acid sequences in woodrats indicates a connection between *CYP2B* genes and the biotransformation of the PSCs in their variable diets. Sequences from the four treatments were not randomly distributed across the ML tree. CYP2B from Great Basin animals fed juniper were differentially distributed across the tree compared to the sequences from the Great Basin animals fed creosote bush and from Mojave animals fed juniper, reflecting both dietary and population-level differences. Visual examination of the tree reveals that Clade A is the only cluster on the tree with representatives from all four treatments (Figure 2). Clades F, G, and H are each dominated by sequences from a different experimental treatment. Clade F is composed primarily of sequences from Great Basin woodrats consuming juniper; Clade G is primarily from Great Basin woodrats ingesting creosote bush, and Clade H mostly Mojave animals ingesting creosote bush. This organization suggests *N. lepida* with different population histories and dietary associations rely on different CYP2B variants to metabolize the PSCs of normal versus novel or ancestral diets.

Genetic variability in protein coding genes is of interest because it provides a mechanistic explanation for differences in protein function, but genetic variability, in itself, is not evidence of difference in protein function. We indirectly tested for functionally important differences by using dN/dS ratios to look for evidence that positive selection was involved in the differentiation of the woodrat CYP2B sequences. If anything, the dN/dS values revealed negative (stabilizing) selection acting to prevent amino acid change across the length of the ORF. The overall dN/dS value was confirmed by the codon-by-codon test for selection, which revealed no evidence of positive selection. This is not entirely surprising, since dN/dS calculations depend on the assumption that the genes in question are from completely distinct lineages, with fully segregated differences. We do not know enough about these sequences to say if they conform to the assumption of segregation required for this test of selection. It is rare, in fact, to find reliable evidence of positive selection for variation within a species, especially for “higher” eukaryotes, such as mammals [56].

Because P450s have been well studied in model systems, we were also able to test potential functional differences in our variants using a more direct, P450-specific approach. Individual amino acid residues of functional importance have been identified from substrate recognition regions in the CYP2B proteins of other species. Changes in the identity of just one of these important amino acids is often associated with changes in the function of the protein [49]. For example, in the black swallowtail butterfly (*Papilio polyxenes*), a single amino acid change (Ile115Leu) in one of these sites in CYP6B1 speeds the metabolism of furanocoumarins, a group of PSCs common in the swallowtail's diet [19]. In the rat, a single amino acid change (Val367Ala) decreases the sensitivity of CYP2B4 to inhibition by 4-phenylimidazole by 7-fold. A change to only 4 of these important residue sites (Ile114Phe, Ser294Thr, Ile363Val and Val367Ala) changes the inhibition sensitivity of CYP2B4 to that of CYP2B5, a different isoform entirely [57].

Using the pharmacological literature for CYP2B, we identified 13 amino acid residues in sites likely to affect substrate recognition. We hypothesized that amino acid changes at these sites would be more likely to result in functional differences than changes in non-substrate recognition residues. Indeed, the distribution of synonymous and nonsynonymous substitutions in these 13 codons was significantly different than substitutions across the rest of the protein. Specifically, at the 13 codons related to substrate recognition, there are a significantly higher proportion of substitutions that result in a change in the amino acid residue. The conflict between the overall dN/dS ratio and the relative increase in nonsynonymous substitutions at the important substrate recognition residues may be a result of selection preserving conserved secondary or tertiary P450 protein structure while allowing for amino acid variation at those few sites important for substrate recognition [48], resulting in functionally unique *CYP2B* variants.

**Figure 3:**
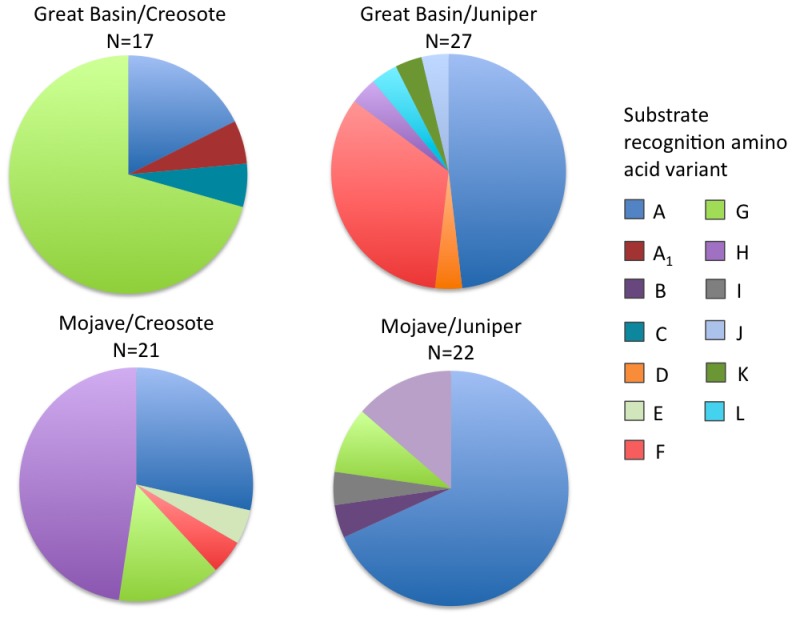
Differential distribution of sequenced *CYP2B* clones across population by dietary treatment. Colors represent groups of sequences with unique combinations of 13 substrate recognition amino acid residues important for CYP2B enzyme function. The smallest wedges in each pie are a unique combination of substrate recognition amino acids that occurs only one time in that treatment. Letters correspond to amino acid characterization in Table 3 and phylogenetic relationships described in Figure 3.

Unique combinations of the 13 substrate recognition residues map to distinct clades on the CYP2B phylogeny (Table 3, Figure 2), suggesting the larger clades on the tree may represent functionally distinct enzymes. For example, all sequences in Clade F (Figure 2), a clade dominated by Great Basin animals fed juniper, share the same amino acid residues across all 13 sites, and this unique combination of residues is not seen elsewhere on the tree. Thus the function of the protein encoded by the Clade F CYP2B may be critical for the biotransformation of juniper. Clade H, which is mostly Mojave Desert animals fed creosote, is more similar to Clade G than to other branches of the tree, but has a different combination of these 13 amino acid residues than Clade G, a clade dominated by Great Basin animals fed creosote. These important amino acid differences may be a result of the Mojave woodrats' unique ecological and evolutionary history with creosote bush. Clade H may be a CYP2B variant similar to that of Clade G, but honed for feeding on creosote bush. Similarly, unique substrate recognition residue combinations are expressed to different degrees by different experimental treatments (Figure 3). For example, all four treatments express the substrate recognition residues unique to Clade A, but juniper-fed animals appear to express relatively more of that variant than creosote-fed animal. Great Basin animals fed creosote and Mojave Desert animals fed creosote each express obviously different patterns of unique substrate recognition variants. Although these patterns are correlational, this is the largest dataset of *CYP2B* messages from a wild herbivore, and it documents a surprising amount of diversity within and across individuals. We cannot tell from our sampling whether the singleton sequences with unique substrate recognition residue combinations (such as B, C, D and E) are allelic variants or rare isoforms. Additional sampling and testing the functional characteristics of these different CYP2B proteins using heterologous expression in a controlled environment is a critical next step in understanding the biotransformation system of wild mammals.

### Conclusions and Future Directions

The activity and efficiency of the mammalian detoxification system has been well studied by the medical and pharmaceutical industries in an effort to understand how human bodies metabolize drugs. However, most of this work has been done in controlled lab conditions using inbred strains of model animals, such as rat and mouse, with limited genetic variability. With these tools, important mechanisms of substrate processing have been documented for many enzymes. However, little is known about how wild animals deal with the wide variety of selective agents confronted by them daily in their diet. The data presented here is the most extensive attempt, to date, to characterize diversity in a detoxification gene of a wild mammalian herbivore.

Wild herbivorous mammals might be predicted to rely more heavily on their detoxification pathways than lab animals, and possess genetic variability reflecting complex genetic and selective dynamics. This study supports the importance of genetic diversity for wild mammalian herbivores. The pattern of CYP2B diversity and expression suggests that the detoxification enzymes of the ancestral desert woodrat were selected to metabolize the wide array of PSCs present in a generalist diet and that this variation was sufficient for the initial switch to a diet of creosote bush. This hypothesis is supported by the lack of a completely unique CYP2B variant specific to Mojave desert animals. However, Mojave desert woodrats are still able to safely consume more creosote than Great Basin animals [30] suggesting differences in the regulatory control of CYP2B variants or in the prevalence of critical variants throughout the Great Basin population. Evidence for this lies in the different distribution of variants across treatments. There might be a selective advantage for an herbivore to maintain little-used but expensive biotransformation machinery, if the promiscuity common to biotransformation enzymes enables the herbivore to metabolize rare or novel toxins in a generalist diet. Indeed, both the Great Basin and Mojave populations of woodrats are generalists; neither can survive on a diet of 100% juniper or creosote bush.

Our exploration of CYP2B diversity in woodrats sets the stage for further comparative work in other herbivores. For example, using the genetic tools developed for *N. lepida*, one can investigate CYP2B in *N. bryanti* Merriam, another woodrat species that also consumes creosote bush foliage, but whose ancestor split from *N. lepida* (1.6 mya) long before creosote bush appeared in North America (10–18 kya) [58]. Ascertaining how these and other more distantly related herbivores (such as *Eucalypus*-feeding marsupials) deal with similar PSCs in their diet will help us compare the roles of regulatory controls versus genetic novelty and convergence versus introgression in the evolutionary process of adapting to a new dietary component. As genomic tools become more advanced and easier to apply to non-model animals (see the panda genome; [59]), these novel, interspecific comparisons will become accessible to ecologists with community- and ecosystem-level questions to answer.

## Supporting Information

Figure S1 **Consensus nucleotide sequence of** ***CYP2B*** **cDNA amplified from desert woodrats (** ***Neotoma lepida*** **).** Underlined sequences are forward and reverse primers (NL_cyp2b_L6; NL_cyp2b_H7, Table 2). Stop and start codons are in bold.
(TIFF)

Figure S2 **Maximum likelihood tree of woodrat** ***CYP2B*** **nucleotide sequences.** Branch length is scaled to the amount of differentiation (see scale bar). Bootstrap support of >50% is indicated on the nodes. Branch labels consist of an individual animal identification number followed by population and diet treatment from the feeding trial. The tree is rooted with the human *CYP2B6* (#AAF32444.1). The nucleotide reconstruction is very similar in topology to the amino acid maximum likelihood tree (Figure 3); the four major clades are identical in sequence membership and similar in both trees.
(TIFF)
