# Supplementary figures and images for: Cytochrome P450 2B Diversity and Dietary Novelty in the Herbivorous, Desert Woodrat (Neotoma lepida)
Source: PLoS One. 2012 Aug 22;7(8):e41510. doi: 10.1371/journal.pone.0041510 (PMC3425548; doi:10.1371/journal.pone.0041510)

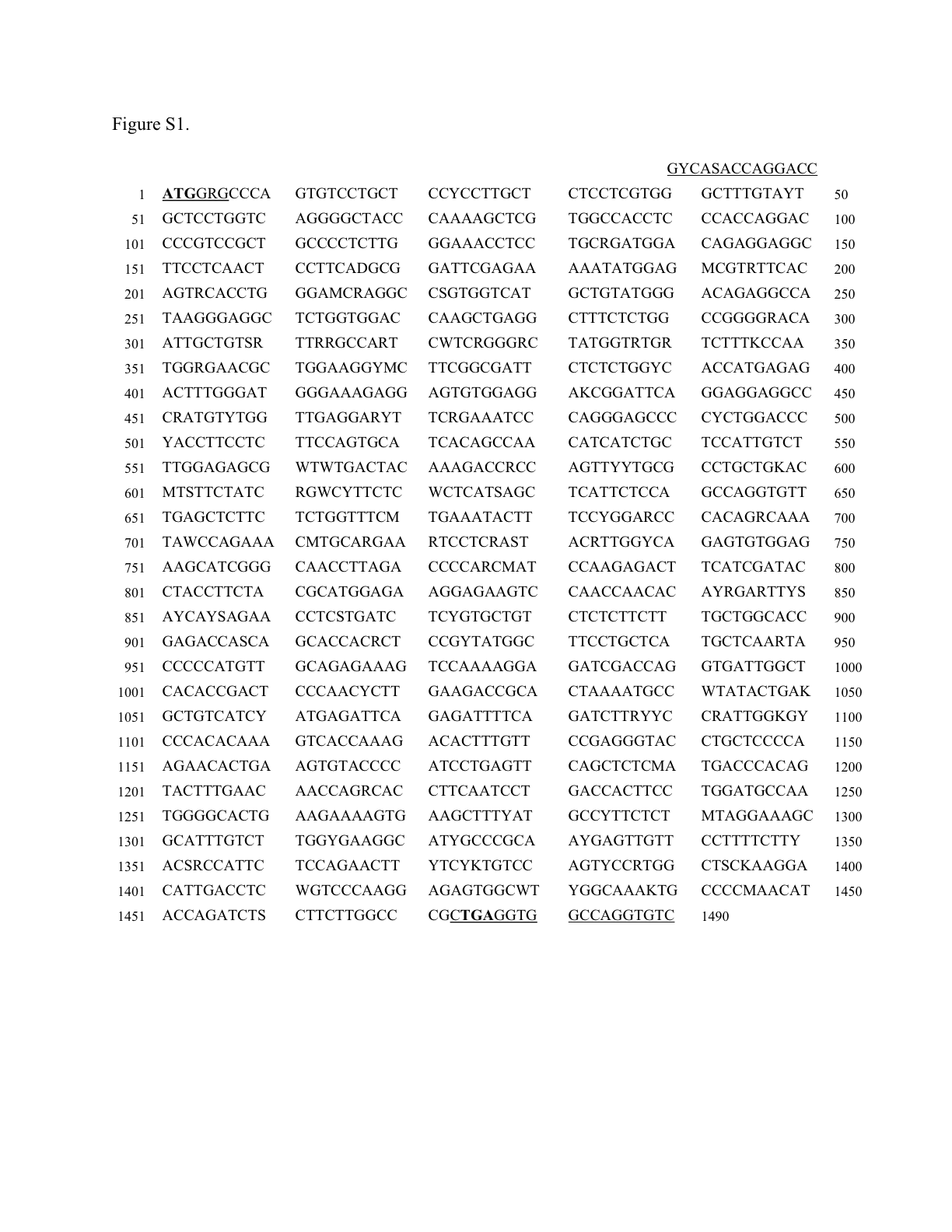

Supplement: Figure S1 — Consensus nucleotide sequence of CYP2B cDNA amplified from desert woodrats ( Neotoma lepida ). Underlined sequences are forward and reverse primers (NL_cyp2b_L6; NL_cyp2b_H7, Table 2). Stop and start codons are in bold. (TIFF) [file pone.0041510.s001.tiff]

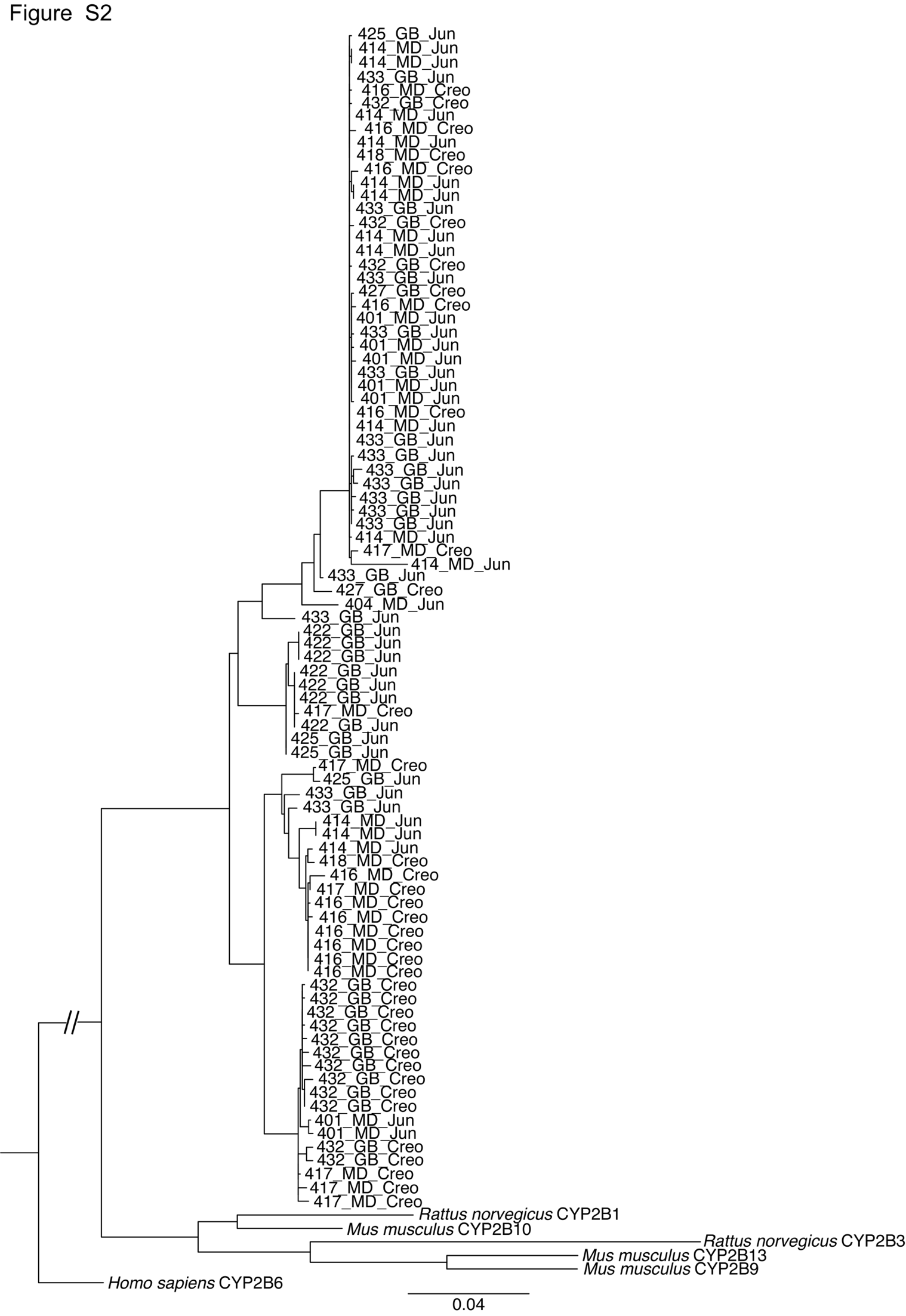

Supplement: Figure S2 — Maximum likelihood tree of woodrat CYP2B nucleotide sequences. Branch length is scaled to the amount of differentiation (see scale bar). Bootstrap support of >50% is indicated on the nodes. Branch labels consist of an individual animal identification number followed by population and diet treatment from the feeding trial. The tree is rooted with the human CYP2B6 (#AAF32444.1). The nucleotide reconstruction is very similar in topology to the amino acid maximum likelihood tree (Figure 3); the four major clades are identical in sequence membership and similar in both trees. (TIFF) [file pone.0041510.s002.tiff]
